# Mishel’s Model of Uncertainty Describing Categories and Subcategories in Fibromyalgia Patients, a Scoping Review

**DOI:** 10.3390/ijerph17113756

**Published:** 2020-05-26

**Authors:** Ana Fernandez-Araque, Julia Gomez-Castro, Andrea Giaquinta-Aranda, Zoraida Verde, Clara Torres-Ortega

**Affiliations:** 1 Department of Nursing, Faculty of Health Sciences, University of Valladolid, 42004 Soria, Spain; juliamaria.gomez@uva.es (J.G.-C.); agaranda1993@hotmail.com (A.G.-A.); clara.torres@enf.uva.es (C.T.-O.); 2 Haemodialysis Service, Santa Bárbara Hospital, 42005 Soria; Spain; 3 Department of Biochemistry, Molecular Biology and Physiology, University of Valladolid, 42004 Soria, Spain; zoraida.verde@uva.es; 4 Emergency Service of the Hospital Santa Bárbara, 42005 Soria, Spain

**Keywords:** fibromyalgia, stigma, illness uncertainty, scoping review, qualitative research

## Abstract

The aim of this review was to demonstrate the presence of categories and subcategories of Mishel’s model in the experiences of patients with fibromyalgia by reviewing qualitative studies. Uncertainty is defined as the inability to determine the meaning of disease-related events. A scoping review of qualitative studies was carried out. Twenty articles were included, with sample sizes ranging from 3 to 58 patients. Articles from different countries and continents were included. Three categories of the model and eight subcategories could be shown to be present in the experiences of fibromyalgia patients through the scoping review. The first category, concerning antecedents of uncertainty in patients with fibromyalgia, is constituted by the difficulty in coping with symptoms, uncertainty about the diagnosis and uncertainty about the complexity of the treatment. The second concerns the cognitive process of anxiety, stress, emotional disorder and social stigma. The third category refers to coping with the disease, through the management of social and family support and the relationship with health care professionals.

## 1. Introduction

Uncertainty characterizes the nature of fibromyalgia syndrome (FMS). FMS is associated with psychiatric comorbidity and coping problems [1,2]. FMS is a chronic pain condition, which has a global mean prevalence of 2.7 % [3,4]. FMS is a chronic musculoskeletal disease which affects physical, mental and sexual health [5]. FMS affects 2.4% of the Spanish population [4]. The Committee of the American College of Rheumatology (ACR) formulated a construct of fibromyalgia 30 years ago in an attempt to rectify a situation of diagnostic confusion faced by patients presenting with widespread pain [6].

Fibromyalgia is a complex syndrome. Evidence-based interdisciplinary guidelines have suggested a comprehensive clinical assessment to avoid this diagnostic conundrum [7]. At diagnosis, most individuals have been experiencing symptoms and have been in the health care system for at least 5 to 8 years [8]. The profound symptomatic impact of FMS may be exacerbated by the perceived inauthenticity [9,10]. Its unpredictable course raises concerns about when and how symptoms will appear or progress, with uncertainty being one of the most characteristic emotions of this disease [11]. All this can have a negative emotional, psychological and socioeconomic impact, both on the patient and on the family [12]. Emphasis in FMS research has shifted over the past decade and in the last year [13]. 

The concept of uncertainty has been used in many disciplines including nursing, medicine, and health communication with slightly differing definitions, extensions, and applications. Nurses provide information that helps patients develop meaning from the illness experience by providing structure. Nurses help patients to manage chronic uncertainty by assisting with patients’ reappraisal of uncertainty from stressful to hopeful in addition to providing relevant information [14,15].

The Uncertainty in Illness Theory and its reconceptualization are models derived from and for nursing practice [16]. Uncertainty in illness is defined as a cognitive state resulting from insufficient cues with which to form a cognitive schema or meaning of a situation or event. Mishel proposes that managing uncertainty is critical to adaptation during illness, and his theory explains how individuals cognitively process illness-associated events and construct meaning from them [17]. This theory is based on Warburton’s information processing theories, the study of Budner’s personality and is influenced in turn by the ideas of Lazarus and Folkman that relate uncertainty with stress and coping [18]. However, Mishel added uncertainty as a stressor in the context of the disease, which is valuable for nurses. This allows us to assess aspects categorized into complex diseases that the individual has to face [19]. The concepts of the theory are organized around three categories using what is known as Mishel’s model of uncertainty in disease [16,17]: firstly, the antecedents of uncertainty refer to the form, composition and structure of the stimuli that the person perceives before some prominent subcategories such as difficulty coping with symptoms, uncertainty regarding diagnosis and uncertainty about the complexity of treatment; the second category is the cognitive appraisal of uncertainty, which can vary from person to person and depends on two main subcategories—uncertainty as a danger, and anxiety, stress expression, emotional disorder and stigma [20]; the final subcategory involves coping with uncertainty through social and family support, through health professionals and through support with peers [21].

Uncertainty affects almost every aspect of a person’s life. In response to the confusion and disorder caused by a state of continuous uncertainty, the system has no choice but to change to survive. Ideally, under conditions of chronic uncertainty, the person should gradually move from a negative assessment of uncertainty to the adoption of a new way of seeing life that accepts this as part of its reality. This occurs through the cognitive assessment of uncertainty and coping with the uncertainty relating to the disease [22,23].

Therefore, we consider it relevant to show how the three basic categories of Mishel’s model for uncertainty about the disease may be present in qualitative studies carried out in patients with fibromyalgia due to its high potential for uncertainty and high emotional demands. Although the studies analyzed in this article are not based on this model, we intend to demonstrate the presence of these categories and be able to detect them, and to show professionals how and where to act, based on the experiences of the patients themselves through the review of qualitative studies. This makes it possible to determine at which moment of the disease process are nursing interventions most time-effective and beneficial for the positive adaptation of the person to their experience of fibromyalgia [24].

The aim of this review was to demonstrate the presence of categories and subcategories of Mishel’s model in the experiences of patients with FMS by reviewing qualitative studies.

## 2. Materials and Methods

A scoping review was carried out, considering qualitative studies with methodological rigor. We selected original articles that explored the experiences of patients with fibromyalgia in order to detect the existence of the three categories present in Mishel’s theory of uncertainty about the disease.

### 2.1. Search

The inclusion criteria for the selection of studies were qualitative designs on adult patients diagnosed with fibromyalgia syndrome, with individual or group intervention methods and verbal accounts of experiences, regardless of the use of the Mishel model as a theoretical framework. Studies selected were conducted within the last ten years, in any language, with free or private access; as we used the library database of the University of Valladolid, this provided us access to articles without free access.

Different search sources and databases were used, such as SCOPUS, CINAHL and PubMed. In the same way, we used descriptors from MESH, CINAHL/MeSH, subject. Headings and DeCS (for the Spanish Language) were employed, in addition to non-standardized terms. The English terms that we used were fibromyalgia, uncertainty, coping, and Mishel. The search was conducted from October 2019 to January 2020, including publications until 2020. We used the following search string for Scopu: fibromyalgia AND uncertainty AND Mishel. For the PubMed database, we used fibromyalgia OR fibro * [tiab] O fibromyalgia AND coping [tiab] O uncertainty [tiab] Mishel O experiences and qualitative investigation [mh]. For the CINAHL Boolean/Phrase, we used the terms fibromyalgia, uncertainty, and Mishel. 

### 2.2. Critical Evaluation

First, we carried out a selection by the title and the abstract of the articles. Then, we discarded repeated articles and those that had no relation to our objective. The articles that we considered relevant, after reading the full text, were evaluated through a peer review. The model used to evaluate the methodological quality of the articles analyzed was the CASPe program for qualitative studies [25], eliminating those that did not meet the minimum established methodological criteria (Table S1). The assessment points included in this guide are “present”, “doubtful” and “not registered”. Therefore, some eligibility criteria were proposed: of the nine questions, when an article presented three negative responses or two negative responses and a doubtful one, this article was excluded for the review of this study. In total, three articles were excluded, which guarantied the methodological quality of this review.

### 2.3. Data Extraction

Subsequently, two authors performed an in-depth reading and extracted the data shown in Table 1. The first extraction consisted of obtaining the following data from each article, for a first analysis: relevant data (author/s, publication date and country); setting context design/model; aim of this study; age range; sample; gender of participants, method of data collection, type of interview.

For the review of the participants’ experiences, the same two authors also proceeded to look for the existence in the discourses of aspects related to uncertainty about the disease itself—called antecedents of uncertainty—with a cognitive appraisal of uncertainty and coping with uncertainty.

The presence of the three categories of the Mishel model allowed us to go one step further by searching for subcategories within each category, which was intended to show real evidence in the verbal accounts of FMS patients with greater emphasis and clarity. Supplementary file 1 shows this review.

### 2.4. Process Followed to Determine the Categories and Subcategories of Uncertainty

We proceeded with two steps to extract and show the presence in the verbal accounts and experiences of patients with FMS of the categories and possible subcategories in each study: (1) we identified statements, responses, individual or group aspects related to uncertainty, cognitive processes and coping with the disease in each study and their coincidence or not with the rest of the studies reviewed; (2) after having identified the categories, we carried out a second in-depth reading that allowed us to obtain subcategories and classify them according to the Mishel model [19].

## 3. Results

**Figure 1:**
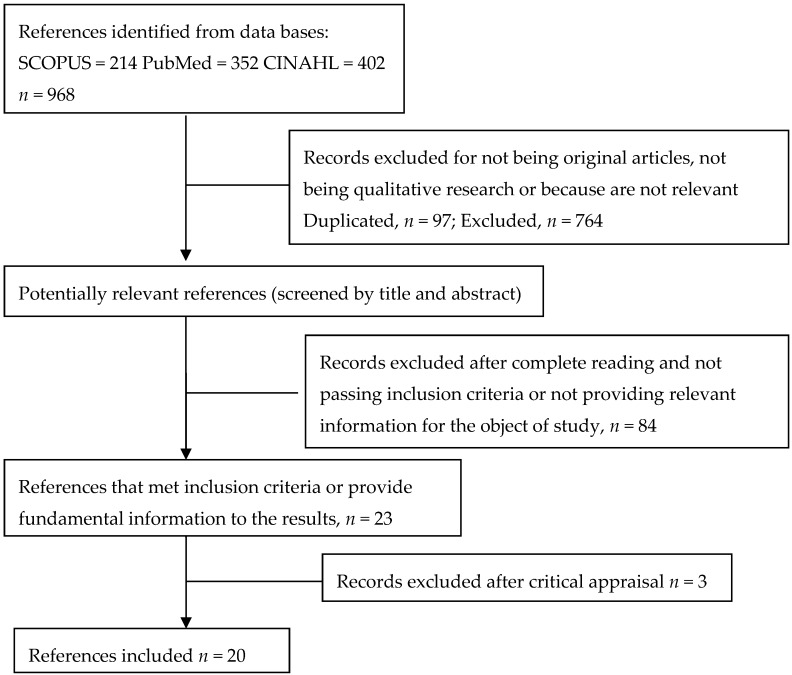
Flowchart of the study selection process.

Our search strategy resulted in a total of 20 final references after the selection process, as observed in Figure 1.

Of the 20 studies that met the inclusion criteria, the sample sizes ranged from 3 to 58 participants. In all studies, the age range was 18 years old and over; the oldest subject was 90 years old. The predominant sex in the studies was female. In total, 45% of the studies reviewed were carried out with women only. Patients were from Canada, the United States, the United Kingdom, Spain, Germany, France, Belgium, Switzerland, Sweden, South Africa, Brazil and Chile (Table 1).

A review of the literature allowed us to identify the existence of categories related to the Mishel model on uncertainty regarding the disease and secondly to group the experiences of fibromyalgia patients by subcategories, obtaining eight relevant subcategories present in the discourses reviewed in the different studies (Table 2, Table S2).

In the first category—antecedents of uncertainty—we integrate the results related to the difficulty in coping with the symptoms, uncertainty about the diagnosis and uncertainty about the complexity of the treatment. The second category of Mishel’s theory is cognitive assessment, which includes anxiety, stress, emotional disorder, and social stigma. The last category refers to coping with uncertainty about the disease. In this case, the subcategories detected in the different discourses and experiences of patients with FMS exhibited a reduction in uncertainty through effective coping due to the existence of subcategories related to the relationship between management and control by health professionals regarding the disease, as well as the support of the family and of peers (Table 2, Table S3).

### 3.1. Antecedents of Uncertainty

#### 3.1.1. Difficulty Coping with Symptoms

In total, 75% of the studies reviewed in our article agree that the unpredictable and changing nature of symptoms leads to a negative evaluation of physical symptoms. The most prominent factors are generalized chronic pain, fatigue, sleep disorders, memory problems, and the feeling of bloating. All these symptoms influence the perceived difficulty to cope with the disease and therefore to adapt to the disease [8,31,33,36]. As a result of the review within this category, we obtained a subcategory—difficulty in coping with symptoms—due to their severity and variability as a stressful factor in the disease process [16,22,28,30,37,40,42] which must be thoroughly evaluated to reduce uncertainty.

#### 3.1.2. Uncertainty Regarding Diagnosis

Diagnosis occurs a last resort because there is no conclusive proven evidence of the disease, so it is considered an invisible disease [27,28]. From the time a patient begins contacting healthcare professionals until diagnosis, an average of approximately 7 years elapses [28,36,41]. During this time, patients are in a situation of uncertainty [40]. In total, 76.16% of the reviewed studies show—in the experiences of FMS patients—the need for a firm diagnosis as a turning point to develop coping strategies in the health–disease process and reduce anxiety. Therefore, it is the second subcategory identified in our results.

#### 3.1.3. Uncertainty about Complexity of Treatment

This third subcategory—the complexity of the treatment of this syndrome—identified appears in the verbal accounts and experiences of 33.33% of the selected studies [27,28,33]. Patients with the highest degree of uncertainty about prognosis were those with the worst adherence to treatment [36]. Most of the participants in the different studies (Table 2) stated that health professionals lack information on the use of the various medications prescribed [40]; patients manifested unpleasant side effects of medications, which occasionally led them to avoid their use [23,28]. These patients then look for other therapy options such as non-pharmacological treatment and moderate physical exercise.

### 3.2. Cognitive Appraisal

In regard to the category of cognitive assessment, the presence of two subcategories is highlighted: the perception of danger by the presence of stress, anxiety and emotional disorders, and the cognitive perception and experience caused by the stigma of the disease [22,29,30,33,36,38,41]. 

#### 3.2.1. Uncertainty as a Danger: Anxiety, Stress and Emotional Disorder

This disease is linked to psychopathology, stress, anxiety and depression. Chronic pain as a routine produces a great deal of suffering and limitations [37], and people affected by FMS feel imprisoned by pain [40]. Another method which some people use to try to confront the diagnosis is to approach religion or spirituality [16], which emerges as an emotional support. Faith in some superior being fills these people with motivation, relief, self-improvement, and strength.

Therefore, stressful and emotional factors may lead to a sense of danger that may constitute another subcategory drawn from the experiences of patients suffering from this disease. Depending on the individuals’ own experiences, education and internal resources, the cognitive assessment of danger will differ from one patient to another. 

#### 3.2.2. Stigma

This subcategory was found in 38% of the selected articles (Table 2). Social stigma is the feeling of prejudice against people with FMS. On one hand, there is a stigma of the mental illness that surrounds this pathology; on the other hand, patients may feel morally offended by the supposed faking of their symptoms, when no obvious signs of illness are found [27,30,33]. Feeling believed by society and supported is a reinforcement of the mechanism of adaptation to FMS [40,41]. Many fibromyalgia patients, in addition to suffering discrimination and prejudice from others, also suffer from these feelings towards themselves.

Furthermore, we discovered another flaw within the social stigma, which we can label as a gender stigma: gender stigma appears as FMS mainly affects women, and there is a stereotype of a “woman who complains” [29]. In total, 14% of the articles analyzed identify gender self-stigma as the inability to fulfil feminine tasks imposed by society, which creates a feeling of remorse in women [29,35,36]. In men, fatigue is seen worse than in women, thus generating a feeling of helplessness and loss of virility [32]. 

### 3.3. Coping with Uncertainty

Coping strategies are the thoughts and behaviors that the person often uses to respond to stressful situations and lessen the perceived threat of experiencing chronic illness [19,20,41]. As a result of our review, we have identified important repetitions in the verbal accounts of the experiences of FMS patients that need to be reduced though effective coping mechanisms.

#### 3.3.1. Coping with Social and Family Support

This subcategory was found in the review by means of three main verbal accounts: social support, family and work repercussion. In 16 of the articles included in this review (Table 2), the importance of sufferers having effective social relationships in their lives is expressed, because these relationships help them to overcome negative situations related to the disease. Pain and relapses can lead to the impossibility of making plans and social isolation, because patients cannot perform the same activities as before the disease [23]. In order not to be judged by people close to them, some patients avoid talking about the disease and the feelings that it entails, therefore avoiding the problem and developing a maladaptation to the situation [41].

Another aspect that many studies show is the great impact on work that the disease produces [32,35,37,40,42]. In environments where there is no public health system, patients may depend on parents or partners to pay for medical bills, leaving those without family support unprotected [31]. It is important to highlight among these results that people try to maintain personal and work roles, using energy dosing strategies to improve their self-concept [39]. The relationship with partners is especially affected because the physical discomfort of these patients leads to a negative mood. On the other hand, the disease involves a change in affectivity and sexual life that is not always well accepted in a couple [29,33,38].

#### 3.3.2. Coping through Health Professionals

One of the subcategories identified and the one that most concerns us is that related to health professionals. This factor was highlighted by more than half of the studies analyzed (Table 2); it was found that a patient’s lack of confidence in healthcare professionals is one of the factors that increases uncertainty. The relationship with healthcare professionals is controversial since there are those who believe in the disease and those who do not [25]. 

Participants demand credibility from healthcare professionals from the first moment they attend the consultation. Empathy is demanded, along with a comprehensive approach, taking into account expectations and the psychological dimension [22,30]. Most often, patients report that their doctors told them the name of their illness, but they were not informed about its possible physical and mental consequences, nor the possible treatments or therapies [27]. This discourse shows us a subcategory that requires the evaluation of our work as health professionals.

#### 3.3.3. Coping through Peer Support (e.g., Associations)

In total, 38% of articles showed that patient associations can play an important role in finding information, especially when the health system is fragile. Patient associations provide peer support, relief, and shared experiences with others in the same situation. For this reason, in our results, this subcategory was detected as an element to contribute to adequate adaptation and an effective method of coping with FMS [40].

## 4. Discussion

The results of this review show the presence of the three main categories of Mishel’s model about uncertainty in adults who suffer from FMS, which was the goal of our study, which was carried out through the revision of the verbal accounts of patients and their experiences in the 20 studies selected. The review included studies from different countries.

This theory has been applied to many chronic diseases, but few studies have been carried out in people with fibromyalgia [11]. However, the identification of uncertainty as a stressor and the ability to establish cognitive processes against stigma and coping through the use and availability of family resources, peers and healthcare professionals can be useful for comprehensive patient care. Furthermore, this can help us to evaluate and help patients to effectively deal with all the aspects related to uncertainty cause by their disease. 

Assessing patients with FMS by these categories will allow us to understand the nature of the uncertainty for each patient individually and will help us to determine how to handle the uncertainty of patients with this disease as these are the most relevant aspects of their disease which concern the patients and interfere with the way they are coping with it [23]. 

The studies do not show differences in uncertainty regarding fibromyalgia between men and women, perhaps due to the lack of studies in men; only one study with men in this review met criteria for inclusion [39], and in those studies that involved men and women, there were a greater proportion of women than men, as can be seen in Table 1 [8,23,35]. Although one author concluded that FMS has a greater impact in men and it causes them to seek medical retirement earlier than women, this has not been proven with a significant sample size or in a qualitative study [43]. 

In regard of patient’s age, the core sample were adults and mainly of intermediate age, which is similar to other studies showing the prevalence of FMS [42]. Patients of working age experience greater uncertainty.

We can affirm the existence of the first category of the Mishel model in the reviewed studies. The perception of patients with FMS, on the basis of initial experience, even before knowing the diagnosis, is similar [4,6,10,34,35,37,43,44], highlighting the difficulties that they have to understand the symptoms, their uncertainty with regard to the diagnosis and uncertainty about the complexity of treatment. It is important to consider the importance of the history of uncertainty in chronic diseases [15,45].

In half of the reviewed studies, we observed that patients mentioned feelings of uncertainty because of the symptoms, because of the pain and diagnosis complexity and because of the lack of adequate treatment [8,27,28,31,32,33,34,37,38,41]. Patients with more pain, who suffer more frequent relapses, have less resistance to stressors related to the disease, and therefore more uncertainty and consequently a worse adaptation [42,43]. All this provokes chronicity and hopelessness [22]. Being diagnosed with fibromyalgia caused mixed feelings in many of the participants [46], although it was a relief for them to have a name for their pain [8,27,30,47]. When patients find a meaning for their disease experience, they reduce their uncertainty or value the knowledge as providing a second chance. This indicates the need to act at this stage and to assess the subcategories of the diagnosis, the symptoms and the treatment, as well as to give information to the patients as nursing professionals and to be in closer contact with the patients, meeting their requirements and supporting them as necessary. 

Other authors such as Boulton et al. [28] and Cedraschi et al. [22] warn that diagnosis does not suffice to reduce uncertainty due to the little knowledge that exists about the evolution of the disease and the prognosis; the feeling of the lack of knowledge of the disease and its treatment floods the affected people with despair. The diagnosis is a relief, although it is sometimes difficult to live with chronicity. The subcategory of the complexity of the treatment showed that those patients with the greatest degree of uncertainty about their prognosis were those with the worst adherence to treatment [36]. This is another important factor that nurses should understand about FMS patients. 

The category of cognitive assessment was reflected in patients with FMS when they reported their experiences of fear and threat [36]. In total, 66% of the patients in the studies analyzed show feelings of vulnerability and fear without really accepting their new health–disease condition. This disease is linked to psychopathology, stress, anxiety and depression. Chronic pain as a routine produces a great deal of suffering and limitations [37], and people affected by FMS feel imprisoned by pain [40]. Another way in which some people try to confront the diagnosis is to approach religion or spirituality [16], which emerges as an emotional support. Faith in some superior being fills these people with motivation, relief, self-improvement, and strength.

Patients use different strategies to cope with the disease, which are family support, peer support, associations and support groups. Health professionals represent a main source of support.

In relation to health professionals, study participants value their credibility from the outset, including their comprehensive and individualized attention. They seek a good level of communication and understanding by professionals, and they need support in the search for effective solutions, considering this therapeutic support in itself [27,30,35]. Therefore, the coordination of different professionals is essential to understand the treatment prescribed, so multidisciplinary sessions would be necessary [27,38]. Multidisciplinary intervention allows the implementation of solutions for self-care, active listening, and reflection, leading to awareness and autonomy [38]. Health education in self-care, support and understanding empowers a person with FMS [32]. According to the search of Alameda et al. [27], the ultimate goal of nursing is to determine the vulnerability caused by the exclusion of these people.

Family members may also experience uncertainty about the illness of one of their relatives; if this occurs, care can be affected. Nurses should address the concerns of caregivers and provide information about the disease [42]. The stability of women improves with the physical and moral support of the partner and family [29].

Therefore, the three subcategories that have been detailed in Table 2 are present frequently in the discourses of the experiences of FMS patients, and these coincide with the most important aspects to assess in chronic and complex diseases [46]. This revision gives credence to these aspects in improving the management of uncertainty.

For some patients, beating FMS means being happy in difficult circumstances and finding small pleasures in daily life [41]. For others, it means clinging to fragments of their previous lives, whether through work or by continuing to pay attention to aspects of daily life. Taking this into consideration, we must continue to carry out studies and reviews that produce a benefit for a better nursing assessment and performance in these patients.

This review is not exempt from limitations. Even though the sources used in the search are pertinent, they might not give a full account of all the relevant studies in this subject. We must bear in mind that, although the databases used to search for the studies are relevant, not all the available studies were collected; therefore, there may be some missing relevant research in this area. We avoided the use of excessive databases to limit the number of repeated articles found; however, a large number of duplicate documents were discarded.

Finally, it would have been interesting to include studies with a more balanced sample of male and female patients and to have been able to review and analyze a gender comparison with more scientific evidence, as well as to have been able to identify possible gender differences and determine actions to further reduce the level of uncertainty and to increase the patients’ ability to cope with the illness.

## 5. Conclusions

The approach to these categories in the assessment of FMS patients can be a valuable tool for a multidisciplinary team since it allows problems to be identified and interventions to be designed which aim to reducing uncertainty and improve patients’ adaptation to the disease.

Uncertainty and FMS are closely linked due to a lack of knowledge about the pathophysiological mechanisms of the disease. 

People diagnosed with FMS often perceive uncertainty as a danger or threat, which leads to situations of stress, anxiety and emotional disorder. The longer the uncertainty lasts in the context of the disease, the more unstable the person’s mode of functioning will be. 

This review demonstrates the presence of three categories and eight subcategories based on Mishel’s model of uncertainty in disease. Addressing these categories can help patients find meaning in FMS, properly manage uncertainty and accept it as a natural part of a new life that makes sense despite the disease.

### Practical Implication

The key message from the study findings for nurses is that they must be alert to potential risk factors for patients with FMS.

This review shows which aspects are most relevant to assess in FMS patients to reduce their uncertainty and face their disease with the best possible professional support.

## Figures and Tables

**Table 1:** Summary of studies included in the review.

Authors, Publication Date and Setting	Context	Design/Theory	Age Range	N/Sample Characteristic	Method of Data Collection	Type of Interview
Alameda et al., 2019 (Madrid, Spain) [26]	Community	Phenomenological	36–74	13/12 W-1 M	Life Story Interview	Individual
Boulton.,2018 (Canada, U.K.) [27]	Community	Narrative analysis	21–69	31/25 W-6 M	In-depth interviews	Individual
Briones et al., 2016 (Valencia, Spain) [28]	Association	Descriptive-exploratory	24–61	13/13 W	In-depth interviews	Individual
Briones et al., 2014 (Valencia, Spain) [29]	Primary care	Descriptive-exploratory	24–61	16/13 W-3 M	Semi-structured interview	Individual
Cedraschi et al.,2013 (Geneva, Switzerland) [22]	Primary care	Interpretative	33–76	56/56 W	Semi-structured interview	Individual
Cooper et al., 2017 (Johannesburg, South Africa) [30]	Community	Descriptive-exploratory	23–59	15/15 W	In-depth interviews and focal group	Individual and group
Escudero-Carretero et al., 2010 (Spain) [8]	Primary care	Interpretative	33–62	21/20 W-1 M	Focal group	Group
Humphrey et al., 2010 (EEUU, Germany, France) [31]	Primary care	Grounded Theory	25–79	40/20/5 M-15 W (EEUU) 10/5 M-5 W(Germany) 10/2 M-8 W (France)	Semi-structured interview	Individual
Juuso et al., 2011 (Sweden) [32]	Community	Phenomenological	38–64	13/13 W	Interview (Unspecified)	Individual
Matarın et al., 2017 (Spain) [33]	Association	Qualitative study with methodology using Gadamer’s philosophical hermeneutics was carried out.	22–56	13/13 W	Focus group and semi-structured interviews	Individual and group
Miranda et al., 2016 (Brazil) [34]	Primary care	Grounded Theory		11/Unspecified	Semi-structured interviews Group dynamics and participant observation	Individual and group
Montesó-Curto et al., 2018 (Catalonia, Spain) [35]	Primary care	Phenomenological	53–69	44/43 W-1 M	Group Problem-Solving Therapy.	Group
Olive et al., 2013 (Cataluña, Spain) [36]	Community	Ethnographic narrative	49–56	3/2 W-1 M	Participant observation and In-depth interviews	Individual
Oliveira et al., 2019 (Río de Janeiro, Brasil) [37]	Community	Interpretative	33–73	12 W	Observation of group dynamics and semi-structured interview	Individual and group
Romero -Alcalá et al., 2019 (Spain, Chile) [38]	Association	Phenomenological and Gadamerian hermeneutics and the Roy adaptation theory	37–53	35 W and their partner	In-depth interviews and focal group	Individual and group
Sallinen et al., 2019 (Oslo, Norway) [39]	Community	Descriptive- exploratory		5/5 M	Life story interviews	Individual
Sorense et al., 2017 (Norway) [40]	Hospital	Hermeneutic analysis	12–19	Unspecified	In-depth interviews	Individual
Taylor et al., 2016 (Virginia, EEUU) [23]	Community	Interpretative	18–64	20/19 W- 1 M and 20 control	Semi-structured interview	Individual
Triviño Martínez et al., 2016 (Alicante, España) [11]	Community	Phenomenological	45–65	14/14 W	Semi-structured interview	Individual
Wuytack et al., 2011 (Gante, Belgium) [41]	Hospital	Exploratory through Husserl’s concept of transcendental subjectivity	36–66	6/6 W	Semi-structured interview	Individual

FMS: fibromyalgia; W: woman, M: men.

**Table 2:** Categories and subcategories of Mishel’s model of uncertainty in fibromyalgia syndrome identified in the experiences of patients with different qualitative studies.

Categories	1. Antecedents of Uncertainty			2. Cognitive Appraisal			3. Coping with Uncertainty	
Subcategories:	1.1. Difficulty with symptoms	1.2. Uncertainty regarding diagnosis	1.3. About complexity of treatment	2.1. As a danger Anxiety, stress expression, emotional disorder	2.2. Stigma	3.1. Coping with social and family support	3.2. Through health professionals	3.3. Through support with peers (e.g., associations)
Alameda et al., 2019 [26]	✓	✓	✓	✓			✓	
Boulton, et al., 2018 [27]	✓	✓					✓	
Briones et al., 2016 [28]		✓	✓	✓	✓		✓	
Briones et al., 2014 [29]		✓		✓	✓	✓	✓	
Cedraschi et al., 2013 [22]	✓		✓	✓	✓		✓	✓
Cooper et al., 2017 [30]	✓	✓		✓				
Escudero-C.et al., 2010 [8]	✓	✓		✓		✓	✓	
Humphrey et al., 2010 [31]	✓	✓						
Juuso et al., 2011 [32]	✓	✓		✓	✓	✓	✓	✓
Matarín et al., 2017 [33]	✓					✓	✓	✓
Miranda et al., 2016 [34]	✓						✓	
Montesó-Curtó et al., 2018 [35]	✓	✓		✓	✓	✓	✓	✓
Olive et al., 2013 [36]	✓	✓	✓	✓		✓	✓	✓
Oliveira JPR et al., 2019 [37]	✓	✓		✓	✓	✓	✓	✓
Romero-Aet al., 2019 [38]						✓	✓	
Sallimen et al., 2019 [39]		✓	✓		✓	✓	✓	
Sorense et al., 2017 [40]	✓		✓	✓		✓		✓
Taylor et al., 2016 [23]	✓	✓		✓	✓	✓		
Triviño Martínez et al., 2016 [11]								
Wuytack et al., 2011 [41]		✓				✓		

## Supplementary Materials

The following are available online at https://www.mdpi.com/1660-4601/17/11/3756/s1, Table S1: Results after a methodological evaluation using CASPe. Table S2: Analysis of the studies included in the review with the most relevant results for each. Table S3. Categories and subcategories of Mishel’s model of Uncertainty in Fibromyalgia Syndrome, identified in the experiences of patients from different qualitative studies.
