# Supplementary material for: Mishel’s Model of Uncertainty Describing Categories and Subcategories in Fibromyalgia Patients, a Scoping Review
Source: Int J Environ Res Public Health. 2020 May 26;17(11):3756. doi: 10.3390/ijerph17113756 (PMC7312620; doi:10.3390/ijerph17113756)
Supplement: Supplementary file 1 [file ijerph-17-03756-s001.pdf]

Table S1. Results after methodological evaluation using CASPe.

**Source (*n* = 23)**	**1**	**2**	**3**	**4**	**5**	**6**	**7**	**8**	**9**	**Decision**
Alameda et al. (2019)	✓	✓	✓	✓	✓	✕	✓	?	✓	INCLUDED
Boulton T (2019)	✓	✓	?	✓	✓	✓	✓	✓	✓	INCLUDED
Briones-Vozmediano et al.(2018)	✓	✓	?	✓	?	✕	✓	?	✓	EXCLUDED
Briones et al. (2016)	✓	✓	?	✓	✓	?	✓	✓	✓	INCLUDED
Briones-Vozmediano et al. (2015)	✓	✓	✓	✓	✓	✓	✓	✓	?	INCLUDED
Cedraschi et al. (2013)	✓	✓	✓	✓	✓	✕	✓	✓	?	INCLUDED
Cooper S et al. (2017)	✓	✓	✓	✓	✓	✓	✓	✓	✓	INCLUDED
Escudero-Carretero et al.(2010)	✓	?	✓	✓	✓	✓	✓	✓	?	INCLUDED
Humphrey L et al. (2010)	✓	✓	✓	✓	✓	✓	✓	?	?	INCLUDED
Juuso et al. (2012)	✓	✓	✓	✓	✓	✓	✓	?	?	INCLUDED
Matarın et al. (2017)	✓	?	✓	✓	✓	✓	✓	?	✓	INCLUDED
Miranda NACG et al. (2016)	✓	✓	✓	✓	✓	?	✓	✓	?	INCLUDED
Montesó-Curto et al. (2018)	✓	✓	✓	✓	✓	✓	✓	?	?	INCLUDED
Olive et al. (2013)	✓	✓	✓	✓	✓	✓	✓	?	✓	INCLUDED
Reich et al. (2006)	✓	✓	✓	✓	✓	✓	✓	?	?	EXCLUDED
Romero –Alcalá et al. (2019)	✓	✓	✓	✓	✓	?	✓	✓	?	INCLUDED
Sallinen et al. (2019)	✓	?	✓	✓	✓	✓	✓	?	✓	INCLUDED
Sorense et al., (2017)	✓	✓	✓	✓	✓	✓	✓	?	?	INCLUDED
Taylor et al., (2016)	✓	✓	✓	✓	✓	✓	✓	?	?	INCLUDED
Theadom et al. (2010)	✓	✓	?	✓	?	?	✓	?	✓	EXCLUDED
Wuytack et al., (2011)	✓	✕	✓	✓	✓	✓	✓	✓	✕	INCLUDED
Triviño Martínez et al.,(2016)	✓	✓	✓	✓	✓	✓	✓	✓	✓	INCLUDED
Oliveira JPR et al., (2019)	✓	✓	✓	✓	✓	✓	✓	✓	✓	INCLUDED

Note 1:” ✓”= Present; “ ? ”= Doubtful; “✕”= Not on record

Note: Items (1–9) of the program CASPe to evaluate the articles methodological quality (Cano Arana et al., 2010).

Source: own elaboration

Table S2. Analysis of the studies included in the review with the most relevant results of each one of them.

Authors, Publication Date and Setting	Context	Design/Model	Age Range	N/Sample Characteristic	Method of Data Collection	Type of Interview	Results to Highlight
Alameda et al., 2019 [26]	Community	Phenome-nological	36–74	13 / 12 W-1 M	Life Story Interview	Individual	Dueling experience, discomfort and psychological [illegible] with stigmatization processes and lifestyle [illegible] relations with more comprehensive health [illegible]
Boulton, et al., 2018 [27]	Community	Narrative analysis	21–69	31 / 25 W-6 M	In-depth interviews	Individual	Participants reflect that the diagnosis of FM is an [illegible] promise because it does not provide definitive [illegible] or legitimacy to their disease experiences.
Briones et al., 2016 [28]	Association	Descriptive-exploratory	24–61	13 / 13 W	In-depth interviews	Individual	-These women feel remorse and frustration at not [illegible] able to continue fulfilling the gender expectation [illegible] caring for others and home.
Briones et al., 2014 [29]	Primary care	Descriptive-exploratory	24–61	16 /13W-3 M	Semi-structured interview	Individual	Four categories detected:<br>-Difficulties in meeting labor demands.<br>-The need for social support in the work environment<br>-The strategies adopted to continue working<br>-The resistance to leave the labor market.
Cedraschi et al., 2013 [22]	Primary care	Interpretative	33–76	56 / 56 W	Semi-structured interview	Individual	Participants expressed decreases in self-esteem [illegible] feelings such as self-esteem or despair.<br>Global perceptions of persistent pain and [illegible] problems.<br>The perceived low control over their lives, as well as [illegible] beliefs that nothing can be done, increases a feeling [illegible] guilt and vulnerability.
Cooper et al., 2017 [30]	Community	Descriptive-exploratory	23–59	15 / 15W	In-depth interviews and focal group	Individual and group	The support of family, couples and partners plays [illegible] integral role in the process of accepting the [illegible] FM.

Authors, Publication Date and Setting	Context	Design/Model	Age Range	N/Sample Characteristic	Method of Data Collection	Type of Interview	Results to Highlight
Escudero-C.et al., 2010 [8]	Primary care	Interpretative	33–62	21 / 20 W-1 M	Focal group	Group	-They describe a difficult experience with symptomatology that can be disabling for everyc activities. -Until they receive the diagnosis, they perce misunderstanding and loneliness. -They deve different coping strategies, such as seeking informat or associating.
Humphrey et al., 2010 [31]	Primary care	Grounded Theory	25–79	40 / EE. UU N 20 / 5 M-15 W; Germany 10 / 5 M-5 W; France 10 / 2 M-8 W.	Semi-structured interview	Individual	-Feeling of fatigue not proportional to the effort ma associated with a feeling of weakness that interfe with motivation and desired activities. -This fatigue prolongs tasks and makes it difficult concentrate, think clearly or remember things. -Most people with FM who participated in this stu experience fatigue and describe it as more severe fatig than normal.
Juuso et al., 2011 [32]	Community	Phenomenological	38–64	13 / 13 W	Interview (Unspecified)	Individual	-For these women, feeling good meant being in contr having power, finding their own rhythm a experiencing feelings of belonging.
Matarín et al., 2017 [33]	Association	Qualitative study with methodology using Gadamer's philosophical hermeneutics was carried out.	22–56	13 /13 W	Focus group and semi-structured interviews	Individual and group	Three themes define the perception of sexuality these women: -Physical impact: don't touch, don't look. -Sexuality and identity: fighting against their loss. -Impact on the relationship: sexuality as a way connecting the couple.

Authors, Publication Date and Setting	Context	Design/Model	Age Range	N/Sample Characteristic	Method of Data Collection	Type of Interview	Results to Highlight
Miranda et al., 2016 [34]	Primary care	Grounded Theory		11 / Unspecified	Semi-structured interviews Group dynamics and participant observation	Individual and group	-Integrative group therapy is a driving force in [illegible] construction and expansion of knowledge ab[illegible] fibromyalgia and empowerment for self-care. This group strategy has proven to be [illegible] interdisciplinary praxis tool that allows the construct[illegible] of networks of Solidarity of attention.
Montesó-Curtó et al., 2018 [35]	Primary care	Phenomenological	53–69	44 / 43 W-1 M	GroupProblem-SolvingTherapy.	Group	Five themes were identified: - Current problems are often related to histori[illegible] trauma. -There are no "one size fits all" solutions. - Fibromyalgia is lifechanging. -Fibromyalgia is widely misunderstood. -Statistically Significant impacts on physi[illegible] psychological and social are described.
Olive et al., 2013 [36]	Community	Ethnographic narrative	49–56	3 / 2 W-1 M	Participant observation and In-depth interviews	Individual	Four factors detected: Stress generated in the waiting time for diagnosis a[illegible] the vital rupture that the disease means The difficulty of sharing with family and friends The conflict with the health system and the limi[illegible] presence of nurses. The interest of staying active at work and personally.
Oliveira JPR et al., 2019 [37]	Community	Interpretative	33–73	12 W	Observation of group dynamics and semi-structured interview	Individual and group	Two categories of the analytical corpus emerged: -Expression of everyday life, as it affects the continu[illegible] physical pain that leads to emotional, psychological a[illegible] distress. -Impact on social, work life FM is a stigmatized disease, which implies implicit l[illegible] of understanding of society.

Authors, Publication Date and Setting	Context	Design/Model	Age Range	N/Sample Characteristic	Method of Data Collection	Type of Interview	Results to Highlight
							The women found in the group of patients a[illegible] professionals a space to talk, understanding, empat[illegible] solidarity and support.
Romero-Aet al., 2019 [38]	Association	Phenomenological and Gadamerian hermeneutics and the Roy adaptation model	37–53	35 W and their partner	In-depth interviews and focal group	Individual and group	Two main themes:<br>-Facing a new sex life "and" resist the loss of sexuality[illegible] the couple ".<br>Improving intimacy, skin-to-skin contact (during ac[illegible] FMS outbreaks), finding new positions, non-coital [illegible] and the use of sex toys can increase female desire a[illegible] help cope with it.
Sallimen et al., 2019 [39]	Community	Descriptive- exploratory		5 / 5 M	Life story interviews	Individual	Two narrative models found: "Adjust life to match [illegible] disease" and "Be imprisoned for pain."<br>Adjusting one's activities can help control sympto[illegible] and support the ability to work in many cases, but [illegible] some patients, the experience of feeling healthy [illegible] painless may be nothing more than a faded memory.
Sorense et al., 2017 [40]	Hospital	Hermeneutic analysis	12–19		In-depth interviews	Individual	Three main themes:<br>-A life with pain and unpleasant body expressions.<br>- An altered emotional well-being.<br>-The struggle to keep up with everyday life.<br>The teenagers described how they fought for norma[illegible] and for not becoming strangers.<br>However, these young people seem to have a stro[illegible] motivation to fight for normalcy.
Taylor et al., 2016 [23]	Community	Interpretative	18–64	20 / 19 W- 1 M and 20 people for control	Semi-structured interview	Individual	This study categorized five recurrent themes:<br>-Experiences of loss.<br>-Feelings of fear and uncertainty.

Authors, Publication Date and Setting	Context	Design/Model	Age Range	N/Sample Characteristic	Method of Data Collection	Type of Interview	Results to Highlight
							-Influence of stress.<br>-Stigmatization of the disease.<br>-Coping through courage.
Triviño Martínez et al., 2016 [11]	Hospital	Exploratory through Husserl's concept of transcendental subjectivity	36–66	6 / 6 W	Semi-structured interview	Individual	The impact of FM on the work and personal life patients, the impact on their future and aspects of soc interaction. Almost all participants had stopp working, leading to feelings of worthlessness and loss identity. The diagnosis was seen as a relief, marking end of a period of uncertainty.
Wuytack et al., 2011 [41]	Community	Phenomenological	45–65	14 / 14 W	Semi-structured interview	Individual	Study patients perceive a high level of uncertainty linked to the<br>- Difficulty in coping with the symptoms.<br>- uncertainty about the diagnosis<br>- Complexity of the treatment.<br>On the other hand, the ability to cope with the disease will be influenced by social support, the relationship with health professionals and the search for help and information by going to patient associations.

FM: fibromyalgia; W: woman, M: men; Scales four

Table S3. Categories and subcategories of Mishel´s Model of Uncertainty in Fibromyalgia Syndrome, identified in the experiences of patients from different qualitative studies.

CATEGORIES AND SUBCATEGORIES TO THE MODEL OF UNCERTAINTY AGAINST MARIE MISHEL'S DISEASE			
	**Uncertainty Background**	**Self-assessment of uncertainty**	**Coping with the disease**

	Difficulty coping with symptoms, uncertainty regarding diagnosis and complexity of treatment	Uncertainty as a danger: Anxiety, stress expression, emotional disorder	Stigma	Coping with social and family support	Coping and information search related to health professionals and with support and search with peers (e.g. associations)	Uncertainty reduction due to effective coping
Alameda et al., 2019 [26]	✓Difficulty diagnosing the disease.<br>✓There are no effective treatments.<br>✓Each medical specialist prescribes a different treatment.	✓ This chronic disease involves a significant loss of skills, professionally and financially, which causes stress, anxiety and sadness.	✓Stigma: It is considered that the person suffers a mental illness and the stigma of moral offense (it is believed that the person is simulating).	✓It affects family and social level, change in family role and social isolation.	✓ Peer support through partnerships is helpful.<br>The relationship with professionals is controversial since there are those who believe in the disease and those who do not.	✓ Peer support and credibility by health workers can empower the person and help them find a new place in the world.
Boulton, et al., 2018 [27]	✓The diagnosis takes time to arrive.<br>✓Symptomatic variability creates uncertainty.				✓The e participants reveal that they trust health professionals, but that instead of answers, the diagnosis of FM is considered a label that reinforces uncertainty because "it only means that we do not know what happens to you and we cannot cure you".	
Briones et al., 2016 [28]	✓Invisibility of the disease due to its difficult diagnosis.<br>✓Gender discrimination as a female problem.<br>✓Uncertainty surrounds the disease, especially with respect to its etiology.<br>✓This lack of knowledge makes	✓ People feel bad about not being able to help with care of the house and dependent children and relatives.	✓ Gender self-stigma, the inability to accomplish traditionally feminine tasks causes them remorse.<br>Prototype of FM patient: woman who complains.	✓Changing the role of caregiver<br>✓They feel a burden for the family. The family has to adapt to the new situation<br>✓Change at emotional level with the couple.<br>✓Physical evil led to a negative mood that influences the relationship. Change in sex life.	✓ Pay attention to sexuality	✓ The Stability of women improves with the physical and moral support of the spouse and family.<br>✓ Nurses should provide information to family members about the disease and the woman's need to delegate tasks.

CATEGORIES AND SUBCATEGORIES TO THE MODEL OF UNCERTAINTY AGAINST MARIE MISHEL'S DISEASE						
	**Uncertainty Background**	**Self-assessment of uncertainty**		**Coping with the disease**		
	**Difficulty coping with symptoms, uncertainty regarding diagnosis and complexity of treatment**	**Uncertainty as a danger: Anxiety, stress expression, emotional disorder**	**Stigma**	**Coping with social and family support**	**Coping and information search related to health professionals and with support and search with peers (e.g. associations)**	**Uncertainty reduction due to effective coping**
	the diagnosis and difficult therapeutic procedures.					
Briones et al., 2014 [29]	✓Difficulty diagnosing the disease, wait for years	✓ Loss in the labor, social and private sphere	✓ Social visibility	✓ Labor affectation Family and social relationship	✓ In comprehended by doctors, given their invisibility, lack of empathy	✓ Patients seek a good level of communication and understanding by professionals, they need support in the search for effective solutions, this support is in itself therapeutic.
Cedraschi et al., 2013 [22]	✓Difficulty coping with symptoms ✓Feeling that there is no cure for this disease	✓ Patients manifest psychological anguish, negativity and despair	✓ Stigma for being a disease with psychological component		✓ The therapist's attitude influence, sometimes the psychological dimension that is affected by the disease is not addressed.	✓Participants value the comprehensive care offered by health professionals who can identify their strengths and weaknesses. ✓This in turn can help build a shared work space and avoid disappointment and helplessness related to chronic refractory pain.

	CATEGORIES AND SUBCATEGORIES TO THE MODEL OF UNCERTAINTY AGAINST MARIE MISHEL'S DISEASE					
	**Uncertainty Background**	**Self-assessment of uncertainty**		**Coping with the disease**		
	**Difficulty coping with symptoms, uncertainty regarding diagnosis and complexity of treatment**	**Uncertainty as a danger: Anxiety, stress expression, emotional disorder**	**Stigma**	**Coping with social and family support**	**Coping and information search related to health professionals and with support and search with peers (e.g. associations)**	**Uncertainty reduction due to effective coping**
Cooper et al., 2017 [30]	✓Unpredictable nature of the symptoms	✓Participants also experienced feelings of despair, rejection and dejection. ✓Specifically when people did not sympathize with nature and gravity of his experience in fibromyalgia.	✓Skepticism and incrudility	✓Society sometimes does not understand the changing nature of the disease. ✓The support of family members is essential to successfully cope with the disease. ✓The inability of families to offer advice and treatments. ✓The family, parents, husband can provide medical insurance, in private healthcare settings. ✓Couples support is very beneficial	✓ The patient associations, the peer support empathy, the understanding favors the adaptation of the person to FM	✓The family support helps to improve the experience of fibromyalgia and properly cope with it. ✓This occurs in a variety of ways. ✓Peer support can facilitate adaptation: Provide information, advice and support to those ✓Empathy, to find a interlocutor who recognize and has experienced the same unexplained symptoms.
Escudero-C.et al., 2010 [8]	✓Uncertainty until diagnosis ✓Difficulty coping with symptoms	✓Until they receive the diagnosis they perceive misunderstanding and loneliness		✓ Family misunderstanding	✓ Participants say they need patient care with more professional training on the disease and training for patient self-care. ✓Empathy, understanding, comprehensive attention with the	✓ They develop different coping strategies, such as seeking information or associating

CATEGORIES AND SUBCATEGORIES TO THE MODEL OF UNCERTAINTY AGAINST MARIE MISHEL'S DISEASE						
	**Uncertainty Background**	**Self-assessment of uncertainty**		**Coping with the disease**		
	**Difficulty coping with symptoms, uncertainty regarding diagnosis and complexity of treatment**	**Uncertainty as a danger: Anxiety, stress expression, emotional disorder**	**Stigma**	**Coping with social and family support**	**Coping and information search related to health professionals and with support and search with peers (e.g. associations)**	**Uncertainty reduction due to effective coping**
					expectations of the person.<br>✓Importance of associations.	
Humphrey et al., 2010 [31]	✓Difficulty coping with symptoms.<br>✓Generalized pain is the main symptom followed by fatigue.		✓Fatigue is worse seen socially in men than in women.			
Juuso et al., 2011 [32]		✓ They develop different coping strategies, such as seeking information or associating	✓ Little social and professional recognition	✓ Importance family support<br>Importance of continuing the daily routine of family care and work, improves self-concept	✓Importance of listening and informing patients by individualizing care by health professionals.	✓Women with FM said they felt good when they could do things at their own pace and avoid stressful situations.<br>✓Through the years they had reached an agreement with the pattern of the disease that allowed them to feel good despite the periods of greatest pain.<br>✓Women with FM felt good when they were heard and understood.
Matarín et al., 2017 [33]	✓Difficulty coping with symptoms			✓ Importance of partner support, the disease affects affective level, affects sexual life with the partner	Patients manifest in relation to this factor:<br>✓ Need more support from professionals<br>✓ Increase information about	✓In their battle to preserve their sexual health, women require support and understanding from their partner, their socio-family environment and health

CATEGORIES AND SUBCATEGORIES TO THE MODEL OF UNCERTAINTY AGAINST MARIE MISHEL'S DISEASE						
	**Uncertainty Background**	**Self-assessment of uncertainty**		**Coping with the disease**		
	**Difficulty coping with symptoms, uncertainty regarding diagnosis and complexity of treatment**	**Uncertainty as a danger: Anxiety, stress expression, emotional disorder**	**Stigma**	**Coping with social and family support**	**Coping and information search related to health professionals and with support and search with peers (e.g. associations)**	**Uncertainty reduction due to effective coping**
					sexuality<br>✓ The importance of peer support and associations	professionals.
Miranda et al., 2016 [34]					✓ Individualized health education, support and understanding empowers the person with FM.<br>✓Benefit of peer support	✓The integrative community therapy group is a driving force in the construction and expansion of knowledge about fibromyalgia and empowerment for self-care
Montesó-Curtó et al., 2018 [35]	✓Severity and unpredictability of symptoms: pain fatigue and overload, memory loss	Anxiety Depression	✓ Misunderstood disease.<br>✓ Gender self-stigma, the inability to accomplish traditionally feminine tasks and not asking the couple for help.	✓ It affects work and social relations Misunderstood by family and friends.	✓ More professional training is needed<br>Introduce the gender perspective.<br>✓ The patients felt misunderstood by the health system: doctors, specialists, being a disease for which little information is available.	✓ Intervention targeting thoughts and emotions of the disease could reduce symptoms and improve pain tolerance and functional status
Olive et al., 2013 [36]	✓Stress generated in the waiting time until diagnosis<br>✓Difficulty living with	Vital rupture that means the disease		✓ Lack of family and social support<br>Need to maintain personal and work	✓ Importance of information by professionals<br><br>✓ Benefit of the associations	✓ The diagnosis is a relief.<br>✓ However the little information that accompanies the disease makes it difficult to handle

CATEGORIES AND SUBCATEGORIES TO THE MODEL OF UNCERTAINTY AGAINST MARIE MISHEL'S DISEASE						
	**Uncertainty Background**	**Self-assessment of uncertainty**		**Coping with the disease**		
	**Difficulty coping with symptoms, uncertainty regarding diagnosis and complexity of treatment**	**Uncertainty as a danger: Anxiety, stress expression, emotional disorder**	**Stigma**	**Coping with social and family support**	**Coping and information search related to health professionals and with support and search with peers (e.g. associations)**	**Uncertainty reduction due to effective coping**
	pain. ✓Symptomatic treatment makes adherence difficult.			roles		uncertainty and move towards harmonious adaptation.
Oliveira JPR et al., 2019 [37]				✓ Importance of partner support ✓Change in affectivity and sex life	✓ Importance of the affectation that the disease has in sexual life.	✓The couple's health education to improve sex life will improve the self-esteem of people affected by FM.
Romero-Aet al., 2019 [38]	✓Uncertainty until diagnosis ✓Side effects of the medication		✓ Medical undervaluation	✓ Gender role involvement Importance of Family Support	✓ Bad relationship doctors Greater understanding nurses ✓ Difficulty finding men with fibromyalgia	✓People should adjust activities to control symptoms and be able to perform social or work activities.
Sallimen et al., 2019 [39]	✓ Difficulty coping with symptoms, imprisoned for pain.	✓ Difficulty coping with symptoms, imprisoned for pain		✓ Decrease social support Risk of isolation and loss of roles		However, these young people seem to have a strong motivation to fight for normalcy, despite
Sorense et al., 2017 [40]	✓There are few objective indicators of the disease. ✓The treatment is complex.	✓ Feelings of fear, stress, when suffering from a chronic illness	✓ Poorly known disease, invisible	✓Loss of roles, independence, work ✓Importance of support from family and friends ✓Inability to make plans.		✓ Diagnosis is a relief. ✓ For these participants, beating FM meant being able to be happy in difficult circumstances, finding small pleasures in their daily

CATEGORIES AND SUBCATEGORIES TO THE MODEL OF UNCERTAINTY AGAINST MARIE MISHEL'S DISEASE						
	**Uncertainty Background**	**Self-assessment of uncertainty**		**Coping with the disease**		
	**Difficulty coping with symptoms, uncertainty regarding diagnosis and complexity of treatment**	**Uncertainty as a danger: Anxiety, stress expression, emotional disorder**	**Stigma**	**Coping with social and family support**	**Coping and information search related to health professionals and with support and search with peers (e.g. associations)**	**Uncertainty reduction due to effective coping**
	✓There is concern about the side effects of the medications. ✓Symptoms are aggravated with adverse, stressful events.					lives. ✓ For others it means clinging to fragments of their previous lives and be it through work or continuous aspects of everyday care.
Taylor et al., 2016 [23]	✓Diagnostic difficulty.			✓ Impact on work, social and family life Feelings of worthlessness when you stop working		
Triviño Martínez et al., 2016 [11]	✓Patients with more pain have less resistance to disease stressors and greater health-related uncertainty. ✓Patients with greater uncertainty show less adherence to the therapeutic. regimen.	✓ Most patients manifest feelings of fear, vulnerability and fear without accepting their new health condition disease.		✓ The greater the social support perceived by the person, the greater their ability to cope and their self-esteem.	✓ The lack of trust of people in professionals increases uncertainty. Demand credibility from the first momento. ✓ Patient associations and peer support can be an element to contribute to harmonious adaptation.	✓ The knowledge and management of the uncertainty theory of M. Mishel by health professionals favors that it can be applied to the assessment and design of interventions that favor adaptation and improve the quality of life.

	CATEGORIES AND SUBCATEGORIES TO THE MODEL OF UNCERTAINTY AGAINST MARIE MISHEL'S DISEASE					
	**Uncertainty Background**	**Self-assessment of uncertainty**		**Coping with the disease**		
	**Difficulty coping with symptoms, uncertainty regarding diagnosis and complexity of treatment**	**Uncertainty as a danger: Anxiety, stress expression, emotional disorder**	**Stigma**	**Coping with social and family support**	**Coping and information search related to health professionals and with support and search with peers (e.g. associations)**	**Uncertainty reduction due to effective coping**
Wuytack et al., 2011 [41]	✓Chronic pain as a routine, which causes them a lot of suffering and limitations	✓ Sadness, anguish, hopelessness, insecurity, fear and anxiety.	✓ Disease loaded with subjectivity. Disbelief of the family, coworkers, invisible disease.	✓ Lack of comprehension Social and family affectation	✓ The interdisciplinary intervention allowed to implement solutions of self-care, active listening, reflection, meaning, which leads to awareness and autonomy	Importance of peer support
